# Effect of Hatch Spacing on the Quality of Inconel 718 Alloy Part

**DOI:** 10.3390/ma17020452

**Published:** 2024-01-18

**Authors:** Yuyi Mao, Yintao Gao, Juan Hu, Xiaodong Shen, Hao Zhou

**Affiliations:** 1 College of Materials Science and Engineering, Nanjing Tech University, Nanjing 211816, China; lwtgzyyx2023@163.com; 2 State Key Laboratory of Materials-Oriented Chemical Engineering, Nanjing Tech University, Nanjing 211816, China; 3 Wuxi Institution of Inspection, Testing and Certification, Wuxi 214028, China; gaoyintao@126.com (Y.G.); hujuan20075050023@163.com (J.H.); 4 Nanjing Institute of Environmental Sciences, Ministry of Ecology and Environment, Nanjing 210042, China

**Keywords:** SLM, hatch spacing, microhardness, wear resistance, porous density, Inconel 718 alloy

## Abstract

Selective laser melting (SLM) has attracted increasing attention all over the world. As an important parameter, hatch spacing, which is the distance between scan lines, however, still needs a more systematic study. In this paper, the relationship between hatch spacing and mechanical properties, including microhardness, wear resistance, and porous density, was studied. The testing results revealed that when hatch spacing decreased, the overlapping rate increased which resulted in an increase in the convection in the molten pool. It led to the formation of pores in the molten pool. However, when hatch spacing was too large, the overlapping zone decreased, while the strength between each welding line was not strong enough. It caused a decrease in the quality of printed parts. Combined with the testing results gained in this work, it can be seen that a 0.06 mm hatch spacing was considered as a relatively optimal condition for part formation under 0.05 μm. Comparison of the morphology of the samples printed under different hatch spacing also confirmed the phenomenon observed here.

## 1. Introduction

In recent years, selective laser melting (SLM), which is known as additive manufacturing (AM), has attracted increasing attention due to its high free-forming ability in the formation process [1]. Firstly, metal powder is put into the supplier tank. The plate in the supplier tank then increases one-layer height according to the parameter set before and the roller pushes the powder onto the workplate for printing. After that, the laser begins to melt the metal powder according to the information on each layer of the designed 3D model. Parameters, like laser power, scan speed, scan strategy, and so on, are all applied according to the parameters set before. After one layer is printed, the plate of the supplier tank raises one layer of height again and the workplate goes down one layer of height accordingly and repeats the previously mentioned printing process until the whole model is finished [2,3].

Due to the many advantages presented by the SLM technology, researchers began to focus their attentions on this technology in different kinds of fields, such as aerospace, biomedical, automotive, and so on. It resulted in a rapid development of SLM in the last few years [4]. Many researchers are now trying to optimize the quality of the printed parts by changing different kinds of parameters. Two main types of process parameters can be classified: laser-related parameters and strategy-related parameters [5]. Laser-related parameters, for example, laser power, scan speed, and laser energy density, have a significant impact on the quality of the printed parts and have been studied quite a lot in the last few years. Moraes and Czekanski [6] used a finite element analysis method to study the impact of laser power on different properties of powder and found that 100 W and 200 W laser power was considered as a relatively optimal input power compared to 400 W laser power. Na et al. [7] studied the relationship between mechanical properties of the pure titanium part and laser power and found that the hardness and strength of the sample increased with the increase in the laser power. Wang et al. [8] focused on the formability, microstructure, and other mechanical properties of AZ61D alloy and found that when laser power was in the range of 85 W to 95 W, the morphology of the printed part was in a smooth and even condition. Scan speed was also studied in recent works. Wu et al. [9] found that scan speed has a significant contribution to printing an almost full-density AlSi10Mg part. Spierings et al. [10] tried to analysis the relationship between laser scan speed and mechanical properties of the Scalmalloy part and found that grain size can be significantly decreased with an increase in the scan speed, while morphology did not show any an obvious change in this process. Nguyen et al. [11] demonstrated that the microstructure and mechanical property of the H13 alloy reached a relatively optimal condition when scan speed was selected at 200 mm/s. Laser power density was also studied by many researchers. Kluczynski et al. [12] studied the influence of energy density on the microhardness of the printed parts. They thought that with an increase in the energy density, the microhardness of the sample also showed an improvement in this process due to the grain boundary strengthening. Lykov [13] tried to compare various energy density parameters in his work by printing different kinds of metals. He found that each mode in SLM process was determined by different process parameters, and he tried to optimize the computational formula of energy density for approximate comparison. Tucho et al. [14] optimized the porosity of the SLMed part and found that when laser energy density was within the range of 50 to 80 J/mm^3^, the porosity decreased with the increase in energy density while the hardness of the sample increased at the same time.

Strategy-related parameters, like scan strategy, layer thickness, and building direction also attracted some attention recently. Jia et al. [15] rotated the scan strategy in the formation process and found that the residual stress reached a relatively optimal condition when the rotation angle reached 15°. Han et al. [16] found that scan strategy had a strong effect on the performance of the surface quality due to the temperature distribution and energy stability in the printing process. According to this finding and the character of the inner structure part, they proposed a new scan strategy which was more suitable to print an inner structure part. Ali et al. [17] used alternating scan strategy to lower the residual stress of the printed part. They found that 90° was the most suitable parameter to print the lowest residual stress sample. The role of layer thickness was also studied by some researchers in different fields. Nguyen et al. [18] thought layer thickness had a great impact on the microstructure of the Inconel 718 sample due to the different cooling rate and crack formation and hardening also related to the layer thickness in the printing process. Ahn [19] considered that the process window was fundamental to forming a high quality part and a single tract test cannot ensure a successful fabrication. Based on these, he proposed a new method for multilayer fabrication. As for the building direction, Dylan et al. [20] found that it had a significant impact on the yield stress with the changing of the building direction, while the samples printed under 45° and 0° were more ductile compared to the part printed under 90°. Xie et al. [21] found that microstructure of the TC4 alloy was highly affected with the changing of the building direction due to the different cooling rate in the formation process which resulted in different tensile performance.

Different from the abovementioned two main kinds of process parameters, hatch spacing, which is both affected by laser-related parameters and strategy-related parameters, was also studied in the last few years. Greco et al. [22] mentioned hatch spacing in their work. They declared that input energy density alone should not be considered as a major factor affecting sample characteristics, but rather the ration of many other process parameters, including hatch spacing. Dong et al. [23] found that hatch spacing was a quite important factor affecting the molten pool behavior of 316 L part in the formation process, and they optimized hatch spacing using a simulation method and validated with experiments. Xia et al. [24] also studied the molten pool behavior in their work on the formation process of Inconel 718 alloy part and found that it had a quite significant impact on the surface roughness of the printed part. Mirkoohi et al. [25] used a three-dimensional temperature prediction model to study the effect of hatch spacing on the molten pool behavior. Accuracy was considered as a major factor in their work and hatch spacing was optimized accordingly.

Considering all the abovementioned works, it can be significantly found that although many researchers admitted the importance of hatch spacing, the effect of hatch spacing on many properties, such as wear resistance, hardness, and porosity, received little attention in recent works compared to laser-related and strategy-related parameters, which resulted in an insufficient understanding of the formation mechanism of this parameter. So, in this work, hatch spacing was systematically researched on the printed Inconel 718 alloy part to study its influence on the wear resistance, hardness, and porosity of the printed parts and a reasonable explanation will also be given accordingly.

## 2. Materials and Instruments

### 2.1. Materials

**Figure 1:**
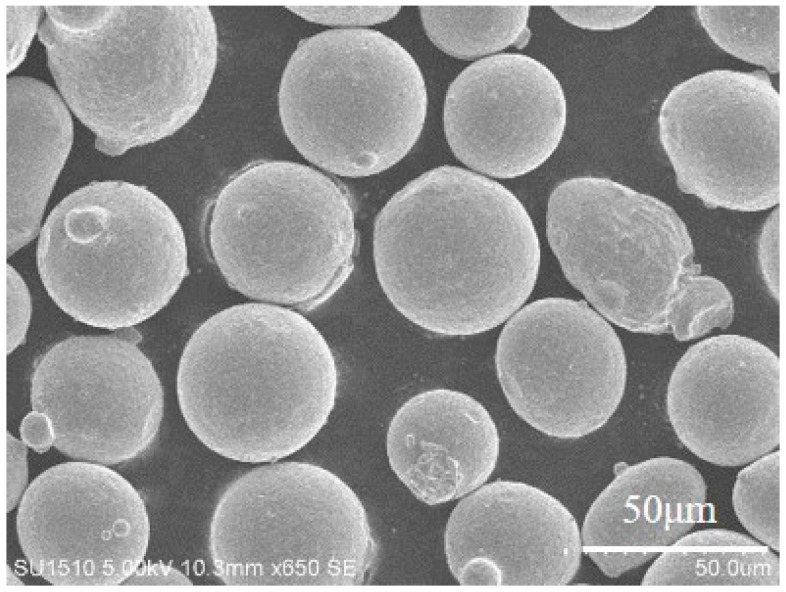
Morphology of the Inconel 718 alloy powder used in this work.

The Inconel 718 alloy powder used in this work was manufactured using gas atomization method which was applied by Falcontech. Co. Ltd., Wuxi, China. The diameter and chemical composition of the powder are shown in Table 1. The microstructure of the powder used in this work is also shown in Figure 1.

### 2.2. Instrument

**Figure 2:**
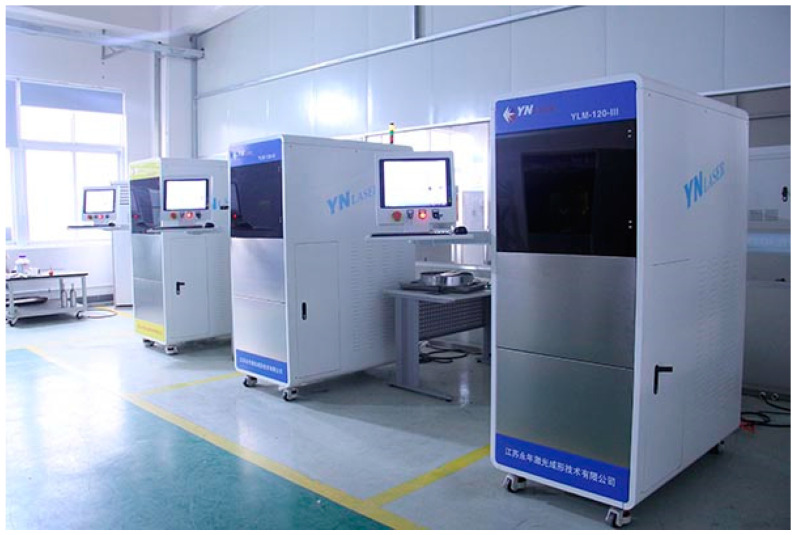
YLM-120 SLM machine used in this work.

The SLM machine used in this work was provided by Jiangsu Yongnian Laser Forming Technology Co., Ltd. (Kunshan, China) and the product of this instrument was YLM-120 which is shown in Figure 2. The laser device used in this instrument was SPI redPOWER fiber type laser while the wavelength was 1060 nm. The minimum size of the laser spot was 40 nm. The scan system used in this instrument was high-speed scan galvanometer with F-theta optical lens and the highest scan speed was 7000 mm/s. The detailed information about the process parameters is shown in Table 2.

**Figure 3:**
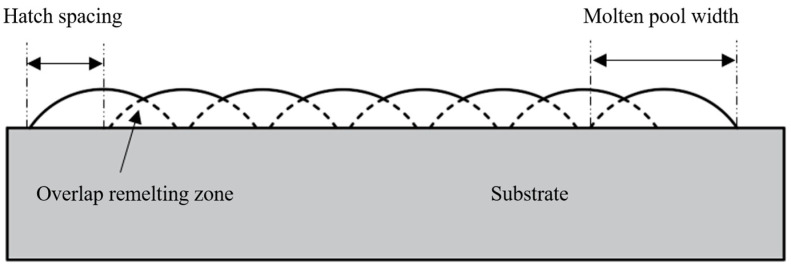
Schematic diagram of the hatch spacing.

**Figure 4:**
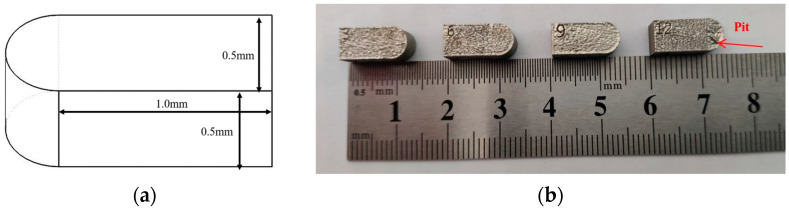
The design sketch (**a**) and printed samples (**b**) used in this work.

Hatch spacing, which is the distance between scan lines, was the only variable parameter in this work and the detailed information is shown in Table 3. The detailed explanation about hatch spacing is shown in Figure 3. The design sketch and the printed samples in this work are shown in Figure 4a,b, respectively.

**Figure 5:**
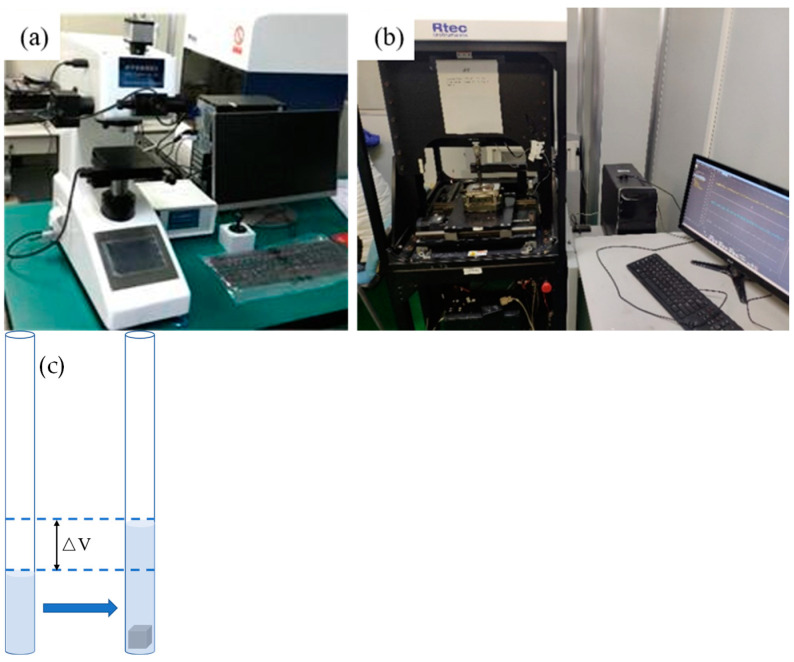
Microhardness tester (**a**), wear testing machine (**b**) and Archimedean Drainage method (**c**) used in this work.

As for the instruments used for testing, two main instruments were employed in this work. One is the semi-automatic digital microhardness tester provided by Shanghai Suoyan Testing Instrument Co., Ltd., Shanghai, China, and its model was HVS-1000ZCM-XYY to test the microhardness value of the printed parts. The wear resistance was tested using the reciprocating friction and wear testing machine provided by RTEC, San Jose, CA, USA, and the model was MFT-5000. As for the porous density of the printed sample, it was mainly measured using the Archimedean Drainage method. All the testing instruments and schematic diagram of Archimedean Drainage method are shown in Figure 5.

**Figure 6:**
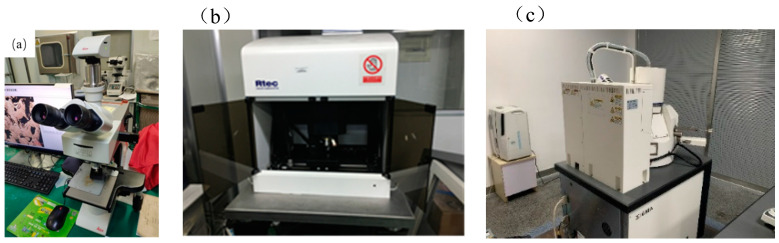
The optical microscope (**a**), white light interferometer (**b**), and scanning electron microscope (**c**) used in this work.

To have a better understanding of the phenomenon gained in this work, optical microscope (OM) DM-2700M, Leica, Germany, and white light interferometer (WLI), RTEC, MFD-D, USA, were used to observe the morphology of the printed parts. A scanning electron microscope (SEM) provided by Carl Zeiss, Sigma 300, Neustadt, Germany, was also used in this work and the images are shown in Figure 6.

## 3. Results and Discussion

### 3.1. Porous Density

The porous density of the samples printed under different hatch spacing was studied first in this work as it caused the least damage on the printed samples compared to other two tests. The Archimedean Drainage method was employed in this work. The volume of the water in the graduated cylinder was labelled as *V*_1_ at first. Then, the printed sample was measured by electronic balance and the weight of the sample was labelled as *M*. After that, we soaked the printed sample in the cylinder and the volume of the water in the cylinder was labelled as *V*_2_. The porous density was then calculated with the following formula.

(1) $$\lambda =\frac{\rho_{1}}{\rho_{2}}\times 100\%=\frac{\left(V_{2}-V_{1}\right)/M}{\rho_{2}}\times 100\%$$

λ was the porous density in this work, while $\rho_{1}$ and $\rho_{2}$ were the printed and ideal state TC4 part density. As porous density was the most significant factor affecting the density of the printed TC4 part, it is quite reasonable to use this formula to calculate the porous density of the printed part. The porous density of the four different samples is shown in Table 4.

From the testing results, it can be clearly seen that the porous density of the sample first decreased with the increase in the hatch spacing. When hatch spacing reached 0.06 mm, the value of the porous density was 99.2% which was relatively optimal in the results gained in this work. With the further increase in the hatch spacing, the porous density was, however, decreased from 99.2% to 98.1%.

**Figure 7:**
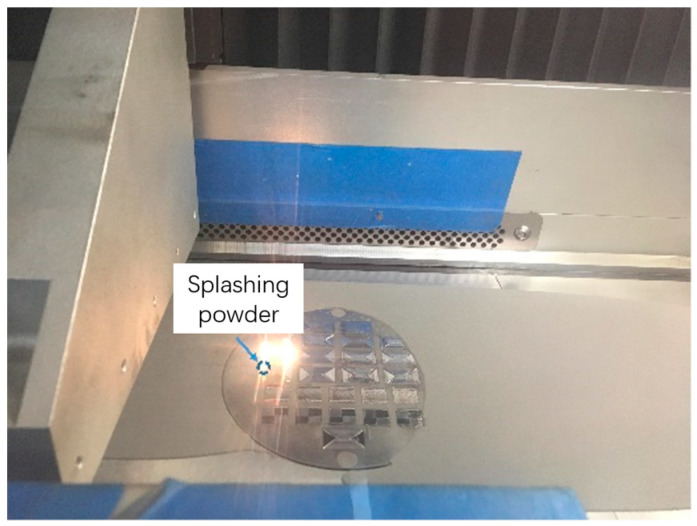
The powder splashing occurred in the printing process.

The reason why the value of porous density was quite small when the hatch spacing was in the range from 0.02 mm to 0.04 mm can be explained by the porous formation in the printing process under a low hatch spacing value. With the decrease in the hatch spacing, the overlapping ratio had an increasing trend. When the overlapping ratio was too high, the heat convection showed a confusion trend. It resulted in the increasing nonuniform temperature distribution of the molten pool which further led to the powder splashing in the printing process on the overlapping zone shown in Figure 7. Powder splashing that happened in the forming process not only affected the quality of the printing layer, but also caused the unevenness of the layer which resulted in a decrease in the overall performance of the printed part.

**Figure 8:**
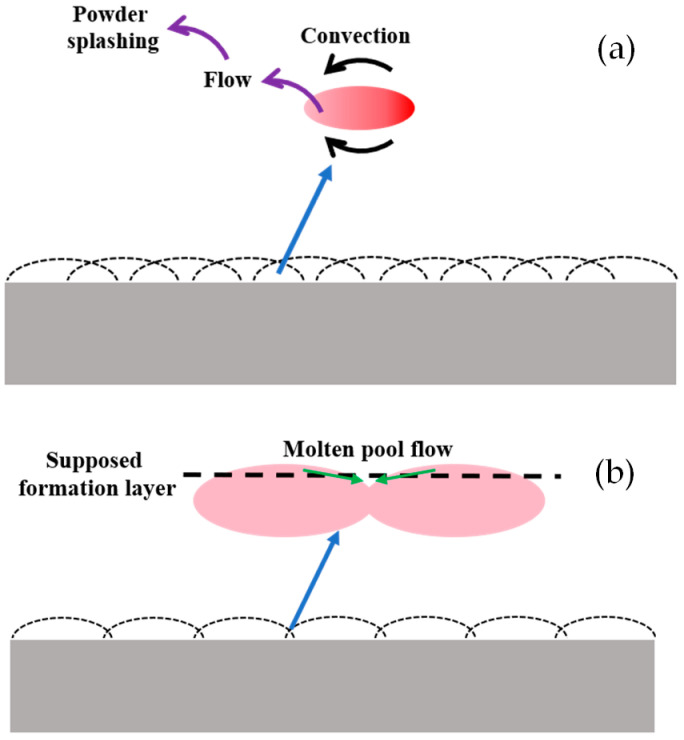
Schematic diagram of the sample printed under small hatch spacing (**a**) and large hatch spacing (**b**), respectively.

Apart from this reason, when the overlapping ratio was too small, the strength between each scan line decreased. Moreover, when the distance between each scan line increased, the molten pool was unlikely to flow to the overlapping zone before its solidification. It caused the formation of large voids in the overlapping zone which decreased the porous density of the printed part. The schematic diagram is shown in Figure 8.

**Figure 9:**
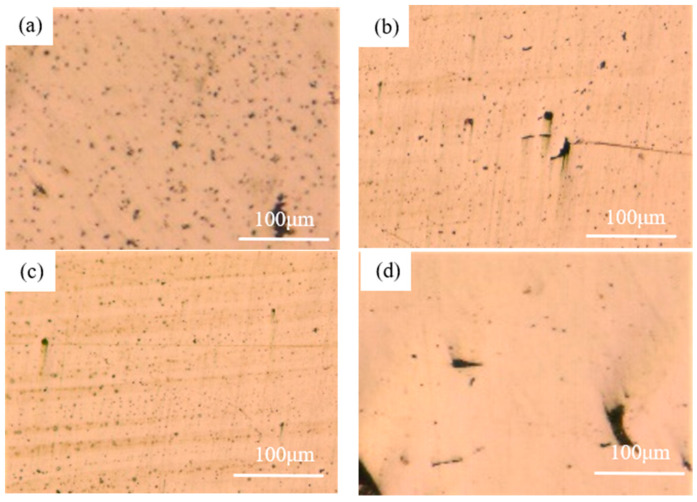
The morphology of the sample printed using 0.02 mm (**a**), 0.04 mm (**b**), 0.06 mm (**c**), and 0.08 mm (**d**), respectively, after polishing.

**Figure 10:**
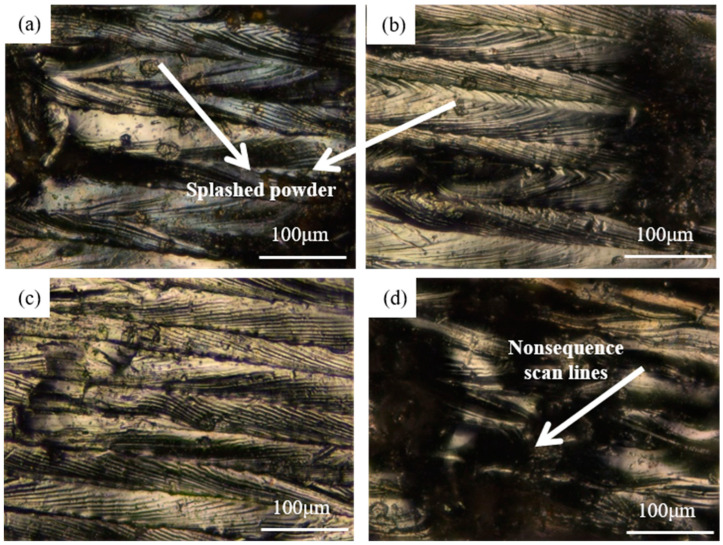
The morphology of the sample printed using 0.02 mm (**a**), 0.04 mm (**b**), 0.06 mm (**c**), and 0.08 mm (**d**), respectively, after printing.

To further verify the explanation given above, the surface morphology of the printed samples was observed by optical microscopy shown in Figure 9. These samples were all meshed using abrasive paper and polishing paste to make it easier to observe the surface morphology. It can be clearly seen that tiny voids can be seen on the samples printed under 0.02 mm and 0.04 mm hatch spacing while some quite obvious voids can be found on the sample printed under 0.08 mm. The results gained here further confirmed the explanation given above. The status of each surface after printing was also observed using OM and is shown in Figure 10. The extra powder bonding can be clearly found on the samples printed under 0.02 mm and 0.04 mm which is in good accordance with the explanation given before.

### 3.2. Microhardness

**Figure 11:**
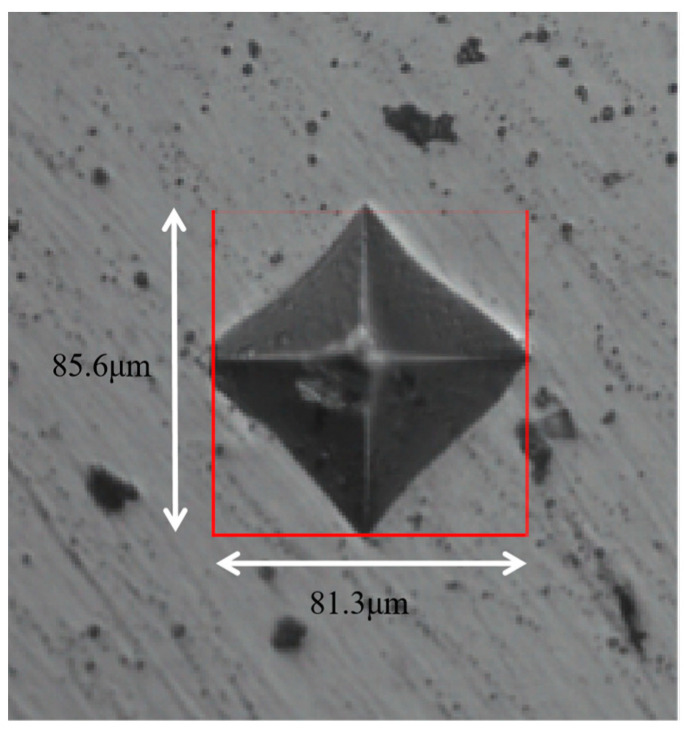
The morphology of the indentation on the surface of the sample.

The microhardness of these four samples was tested using the microhardness tester. The hardness value used in this work was Vickers hardness and the morphology of the indentation was diamond shape as shown in Figure 11. In the testing process, the pressure was kept at 200 N for 12 s and we chose five places for testing randomly. The values which had a large deviation were deleted, and we calculated the average value using the rest of the data, and the measured data are shown in Table 5.

From the testing results, it can be clearly seen that the sample printed under 0.06 mm hatch spacing showed the relatively optimal condition in all of these four samples. The phenomenon observed here was in good accordance with the porous density observed before. With the increase in the porous density of the printed part, the overall hardness had a decreasing trend as illustrated by previous works.

### 3.3. Wear Resistance

Wear resistance of the printed sample was also studied in this work tested by a friction wear testing machine. The normal pressure used here was 10 N, while the frequency of reciprocating motion module was 1 Hz. Taking the substrate material type into consideration, the grinding ball used in this work was a 304 stainless steel ball with a diameter of 3.262 mm. The experimental time was 600 s. The friction coefficient curve was exported directly, and the wear rate can be calculated with the following formula.

$$W=\frac{V}{L\cdot N}$$

$$V=\left(R^{2}\sin^{-1}\frac{D}{2R}-\frac{D\left(R-H\right)}{2}\right)\cdot Z$$

In this formula, *V* represented the friction volume, *R* represented the diameter of the grinding ball, *D* represented the width of the indentation, *H* represented the maximum depth of the indentation, *Z* represented the length of the indentation, *W* represented the friction ratio, *L* represented the distance of the friction, and *N* represented the normal pressure.

**Figure 12:**
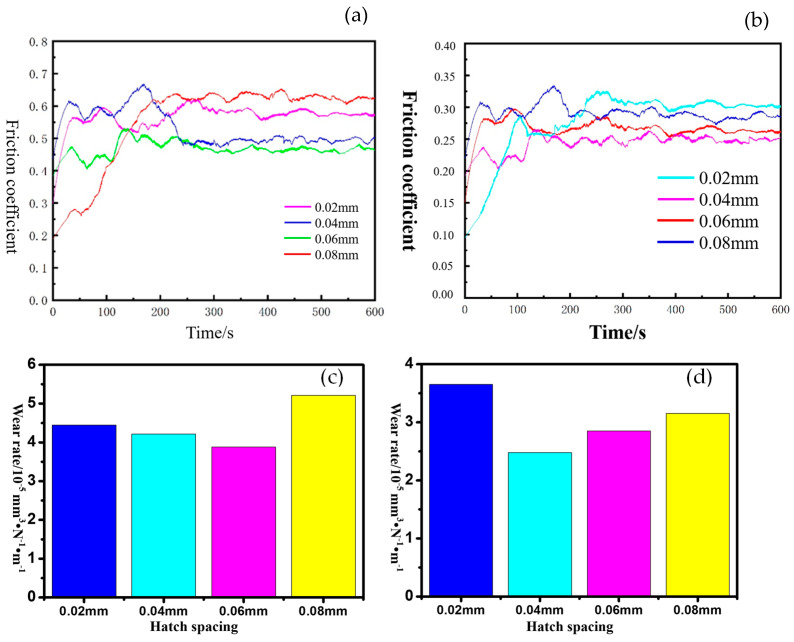
The friction coefficient and wear rate of the part tested in solid-state atmosphere (**a**,**c**) and liquid-state atmosphere (**b**,**d**).

To have a better understanding of the wear resistance of the printed sample, the sample was grinded in solid-state atmosphere and liquid-state atmosphere and the friction coefficient curve of the samples are shown in Figure 12a,b, respectively. Wear rates were also studied in this work and are shown in Figure 12c,d.

The testing results shown in Figure 12 revealed that in the solid-state atmosphere, the sample printed using 0.06 mm hatch spacing showed the best wear resistance in all of these four samples. It was mainly caused by the relatively lower hardness and looser state of the printed part. However, an interesting point can be seen with the wear resistance of the samples soaked in liquid-state atmosphere. Sample 3, which was printed under 0.06 mm hatch spacing, ranked second in the liquid-state atmosphere while the sample printed under 0.04 mm hatch spacing showed the best performance in all of these four samples.

**Figure 13:**
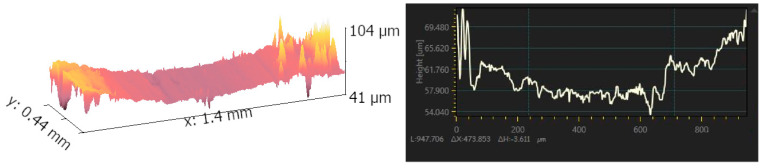
Morphology of the surface after wear and its scratch-depth variation.

This phenomenon was quite different from the results gained before. It was mainly caused by the lubrication effect of the liquid stored inside the voids on the surface. When the grinding ball squeezed the surface in the friction process, the liquid inside the voids squeezed out and acted as lubricant. It can, of course, increase the wear resistance of the printed parts. However, when the size and amount of voids went too high, it also had a significant negative impact on the wear resistance of the printed part. One reason is the loose state of the printed part when too many voids formed in this process. Another reason is that the edge of the voids with a relatively large size was more likely impacted by the grinding ball which resulted in the extra removal of the material. Morphology of the surface after grinding is shown in Figure 13. It can be seen that a furrow-shaped scratch was quite clear in this image and the depth of the scratch also confirmed the observation before.

To sum it up, hatch spacing had a significant impact on the overlapping ratio under the same spot size, and the performance of the molten pool in the overlapping zone showed a different impact under different overlapping ratios. An unsuitable hatch spacing likely led to the formation of the extra voids in this process which caused the decrease in the microhardness and wear resistance in a solid-state atmosphere. However, wear resistance in liquid-state atmosphere showed a different variation trend due to the fact that some voids on the surface can provide a lubrication effect while too many voids or too large voids also decrease the wear resistance even in the liquid-state atmosphere.

## 4. Conclusions

In this work, hatch spacing was systematically researched to study its impact on the mechanical properties of the printed Inconel 718 alloy part, including microhardness, wear resistance, and porous density. The testing results revealed that hatch spacing had a significant impact on the property of the printed parts and 0.06 mm hatch spacing was considered to be a relatively optimal parameter for printing in this work. Detailed conclusions were drawn as follows.

(1) Hatch spacing had a great impact on the overlapping ratio which affected the molten pool behavior in the solidification process. Too large an overlapping zone likely resulted in the random flow of the molten pool which led to the formation of pores in this process. Too small an overlapping zone, however, resulted in the decrease in the bonding strength between the scan lines which also affected the mechanical property of the printed parts.
(2) The formation of pores resulted in the decrease in the hardness due to the relatively loose state of the printed part which led to the decrease in the microhardness. Additionally, the morphology of the printed surface also had an impact on the microhardness of the printed part.
(3) The wear resistance had a different performance in liquid-state atmosphere and solid-state atmosphere. The friction coefficient of the sample printed under 0.06 mm hatch spacing was the lowest in the solid-state atmosphere, while it ranked the second in the liquid-state atmosphere. This was mainly caused by the pores on the surface of the sample.

## Figures and Tables

**Table 1:** Relative information about the Inconel 718 alloy powder used in this work.

Chemical Composition /wt.%	Cr	Fe	Nb	Mo	Ti	Al	Co
	18.93	17.32	5.18	3.08	0.92	0.47	0.041
	Cu	C	Si	Mn	P	S	Ni
	0.058	0.05	0.056	0.13	0.0058	0.0038	Bal
Diameter/μm	D10		D50			D90	
	22.3		33.2			50.4	

**Table 2:** Process parameters used in this work.

Process Parameters	Laser Power	Scan Speed	Layer Thickness	Defocusing Amount	Scan Strategy	Protective Gas	Building Direction
Value	200 W	1000 mm/s	70 μm	0.0 mm	Zigzag strategy	Argon	Y-axis

**Table 3:** Hatch spacing used in this work.

Sample	Sample 1	Sample 2	Sample 3	Sample 4
Value	0.04 mm	0.06 mm	0.08 mm	0.10 mm

**Table 4:** The porous density of the samples printed under different hatch spacing.

Hatch Spacing/mm	0.02	0.04	0.06	0.08
Porous density/%	97.4	98.3	99.2	98.1

**Table 5:** Microhardness of the samples printed under different hatch spacing.

Hatch Spacing/mm	0.02	0.04	0.06	0.08
Microhardness/HV	356	369	388	378

## Data Availability

Data are contained within the article.
